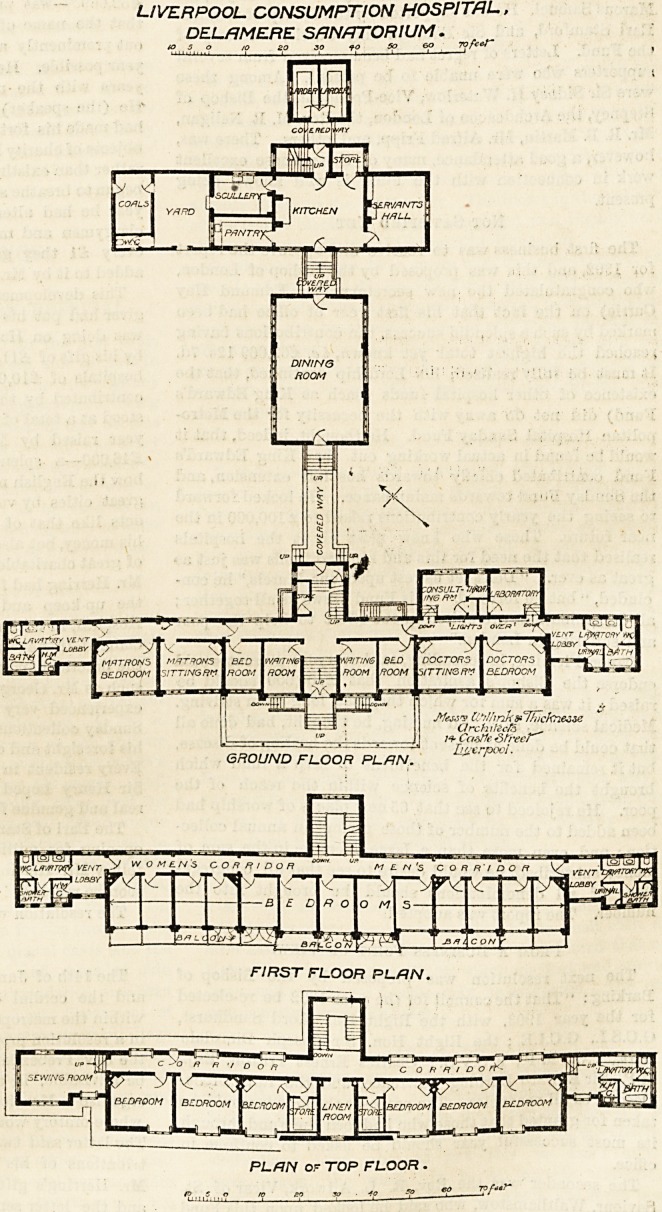# Liverpool Hospital for Consumptives at Delamere Forest

**Published:** 1902-12-13

**Authors:** 


### The Institutional Workshop.

### LIVERPOOL HOSPITAL FOR CONSUMPTIVES AT DELAMERE FOREST.

As will at a glance be seen from the plans we publish herewith, the general arrangement of the component parts of this sanatorium is somewhat in the form of the letter "T," with the top part of the letter facing the south-west. On the ground floor of this section are placed the hall and waiting-rooms, with the matron's rooms towards the west, and the resident medical officer's rooms towards the east end. Behind all these runs a straight corridor which connects the various rooms, and immediately behind the medical officer's rooms are the consulting-room and the laboratory. In the centre to the front is a large glass-covered verandah for the patients' use, and at either end of the block are sanitary annexes. These are properly cut off by cross-ventilated passages; but a few feet of extra space in the annexes themselves would have been highly desirable, and it would have been a better arrangement if the annexes had been placed at an obtuse angle to the main building instead of being in line with it. At the centre of the block to the north is the main staircase, and a covered way connects with the dining hall, beyond which are the kitchen, servants' hall, and other offices.

On the first floor of the main block are the patients' bed-rooms and all these open on to balconies. The top floor provides accommodation for the nursing staff. The sanitary annexes are continued on all the floors except at the extreme west corner of the top floor in which the space is devoted to a sewing-room. We doubt whether a sewing-room placed on the top floor can be convenient, and we rather dislike the idea of placing even a sewing-room over closets and bath rooms. But we are satisfied that the general conception of the design is extremely good; and the correct principle of not housing too many patients under one roof has been adopted. There are five bungalows on the grounds and each of these gives accommodation for four patients. These bungalows might be multiplied almost indefinitely, and will be as occasion requires.

Electric power is generated in a small block placed northwards of the kitchen, and is used not only for lighting purposes, but for pumping the water, and for working the refrigerator in the cold store. The building cost £15,000, and was defrayed by Lady Willox and Mr. W. P. Hartley.

LIVERPOOL CONSUMPTION HOSPITAL, DELAMERE SANATORIUM.

The architects were Messrs. Willink and Thicknesse, of Liverpool.

## Figures and Tables

**Figure f1:**